# Unraveling the multitarget mechanisms of aloe emodin against periodontitis: a synergy of network pharmacology and experimental validation

**DOI:** 10.3389/fphar.2026.1944429

**Published:** 2026-10-20

**Authors:** Yifan Zhang, Huijing Ma, Shengxiang Zhang, Debao Jing, Hao Liang, Yufeng Wang, Zhengrui Li, Huijiao Yan

**Affiliations:** 1 Department of Stomatology, Gongli Hospital of Shanghai Pudong New Area, Shanghai, China; 2 Department of Oral Mucosal Diseases, Shanghai Ninth People’s Hospital, Shanghai Jiao Tong University School of Medicine, Shanghai, China; 3 Shanghai Jiao Tong University School of Medicine, Shanghai, China; 4 Department of Stomatology, Oromaxillofacial Head and Neck Surgery, Huashan Hospital, Fudan University, Shanghai, China; 5 College of Basic Medical Science, Yunnan University of Chinese Medicine, Kunming, China

**Keywords:** aloe emodin, network pharmacology, periodontitis, PI3K/Akt signaling pathway, traditional medicine

## Abstract

**Background:**

Chronic periodontitis is a prevalent inflammatory disease that leads to destruction of periodontal tissues, resulting in tooth loss. While mechanical debridement and adjunctive antibiotics are commonly used, their therapeutic efficacy is limited by anatomical complexity and concerns over antibiotic resistance and side effects. Aloe-emodin (AE), a naturally occurring anthraquinone compound, exhibits significant anti-inflammatory and antimicrobial effects. However, its mechanisms in periodontitis remain unclear.

**Methods:**

A network pharmacology approach was employed to identify potential targets of AE in periodontitis. The effects of AE on the viability, proliferation, migration, osteogenic differentiation, and inflammatory responses of mouse periodontal ligament stem cells (mPDLSCs) were assessed CCK8, qPCR and ELISA. *In vivo*, the alveolar bone of mouse periodontitis model was repaired and evaluated by micro-CT and histological analysis.

**Results:**

Network pharmacology analysis identified 179 overlapping targets between AE and periodontitis, with key hubs including TNF, AKT1, EGFR, TP53, and MMP9. These targets were predominantly enriched in the MAPK, PI3K-AKT, NF-κB, and TNF signaling pathways. *In vitro*, AE significantly enhanced the proliferation, migration, and osteogenic differentiation of mPDLSCs while markedly suppressing LPS-induced expression. *In vivo*, AE dose-dependently reduced alveolar bone loss, improved trabecular microarchitecture, increased bone mineral density, and enhanced collagen organization in the periodontitis model.

**Conclusion:**

AE is a pleiotropic natural compound that reprograms the inflammatory-osteolytic microenvironment of periodontitis and promotes alveolar bone regeneration through multi-target pathway modulation.

## 1 Introduction

Chronic periodontitis is a persistent inflammatory disease of the periodontal supporting tissues initiated by plaque biofilm microorganisms (Tonetti, 2018). Early manifestations include gingival erythema, edema, and bleeding. With progression, inflammation spreads from the gingiva to the periodontal ligament and alveolar bone, leading to attachment loss, tooth mobility, and ultimately tooth loss, with substantial deterioration in quality of life (Hajishengallis and Chavakis, 2021). Epidemiological surveys indicate that periodontitis remains one of the most prevalent oral diseases worldwide and a leading cause of tooth loss in adults. Standard care relies on mechanical debridement (ultrasonic and/or hand instrumentation) to control subgingival biofilm; however, complex root anatomy and inaccessible niches can limit instrumentation and reduce clinical effectiveness (Balta et al., 2021). Consequently, adjunctive antimicrobials such as metronidazole and tetracyclines are frequently used (Caivano et al., 2025). Prolonged antibiotic exposure may induce oral microbial dysbiosis and cause adverse events, including gastrointestinal symptoms and systemic toxicity (Lu et al., 2025). Given favorable safety profiles and therapeutic potential, natural compounds merit further exploration as adjuncts for periodontitis management.

Aloe-emodin is a naturally occurring anthraquinone found mainly in the roots and rhizomes of medicinal plants including Aloe vera, Cassia occidentalis, and Polygonum multiflorum Thunb (Ren and Wesseling, 2024). AE exhibits diverse bioactivities—anticancer, antiviral, antibacterial, immunomodulatory, and hepatoprotective effects—supporting its relevance to disease prevention and treatment (Chen et al., 2025; Shang et al., 2022). Anti-inflammatory activity has been linked to inhibition of NF-κB, MAPK, and PI3K signaling and reduced release of pro-inflammatory cytokines (Otieno et al., 2021). In oral medicine, AE attenuates periodontitis-associated alveolar bone resorption and suppresses Porphyromonas gingivalis growth (Yang et al., 2022), highlighting potential utility for periodontal diseases. As a bioactive, low-toxicity compound, AE may offer value in periodontitis therapy, especially amid increasing antibiotic resistance (Nowak-Perlak et al., 2025). Nonetheless, the molecular mechanisms by which AE regulates periodontal inflammation remain insufficiently defined.

Network pharmacology integrates bioinformatics and systems biology to construct “drug–active ingredient–target” networks from high-throughput omics and computational analyses, enabling systematic characterization of drug–host interactions (Zhu et al., 2023). This framework is widely applied to traditional Chinese medicine research. Coupling large-scale data mining and target prediction with molecular docking to validate key ligand–target interactions, network pharmacology can clarify disease mechanisms, inform therapeutic strategies for complex disorders, and provide a systems-level view of drug–disease relationships (Merecz-Sadowska et al., 2025).

This study applied network pharmacology to elucidate the molecular basis of AE against periodontitis. Core targets were prioritized for molecular docking to evaluate binding affinity between AE and candidate proteins. Combined with *in vitro* and *in vivo* validation, this study assessed anti-inflammatory and osteogenic effects of AE, providing a mechanistic rationale for developing novel therapeutics for chronic periodontitis.

## 2 Materials and methods

### 2.1 Network pharmacology analysis

Potential AE targets were identified through a multi-database workflow. The AE structure was analyzed by inverse docking and similarity-based target prediction using PharmMapper, the SEA Search Server, and the Herbal Ingredients’ Targets (HIT) platform. Predicted targets were standardized in UniProt (*Homo sapiens*), yielding 290 non-redundant AE-associated targets.

Periodontitis-related genes were collected from GeneCards, OMIM, and the Comparative Toxicogenomics Database (CTD) using the term “periodontitis.” After integration and removal of duplicates, 4,609 disease-associated targets were retained. Overlap analysis between AE- and periodontitis-related targets defined candidate therapeutic targets.

Shared targets were uploaded to STRING (*Homo sapiens*; confidence score ≥0.4) to generate a protein–protein interaction (PPI) network. The network was visualized in Cytoscape, and topological parameters were computed with CytoNCA; hub targets were prioritized by node degree. Functional clusters were further extracted using MCODE.

Gene Ontology (GO) enrichment and KEGG pathway analyses were performed on the shared targets to characterize implicated biological processes and signaling pathways. Enrichment outputs were presented as bar plots, bubble plots, circular plots, and Sankey diagrams.

AE–Target–Disease and AE–Target–Pathway networks were constructed in Cytoscape to depict the multi-target, multi-pathway basis of AE activity against periodontitis.

### 2.2 Cell culture

Primary mouse periodontal ligament stem cells (mPDLSCs; Hunan Fenghui Biotechnology Co., Ltd., No. FHMIC2086) were cultured in α-MEM supplemented with 10% fetal bovine serum (FBS) and 1% penicillin–streptomycin at 37 °C in a humidified 5% CO_2_ atmosphere. Cells at passages 3–6 were used. AE (TCI Chemicals, Shanghai, China) was dissolved in DMSO and diluted in culture medium to the required concentrations.

### 2.3 Cell viability assay

Cytotoxicity and the working concentration range of AE were determined using a Cell Counting Kit-8 (CCK-8) assay (Dojindo, Japan). mPDLSCs were seeded in 96-well plates (5 × 10^3^ cells/well) and exposed to AE (0, 5, 10, 15, and 20 μM) for 24, 48, or 72 h. At each time point, 10 μL CCK-8 reagent was added per well and incubated for 2 h at 37 °C. Absorbance was measured at 450 nm (Bio-Rad, United States of America), and viability was calculated relative to untreated controls. Concentrations >20 μM were excluded because preliminary cytocompatibility screening and prior evidence indicate an increased likelihood of non-specific cellular stress responses at higher doses. Accordingly, ≤20 μM was used to preserve biological relevance and experimental robustness. The lowest effective concentration was selected to minimize off-target effects and better approximate clinically relevant exposure.

### 2.4 Colony formation assay

To assess clonogenic potential, mPDLSCs were seeded in 6-well plates (500 cells/well) and exposed to the optimized AE concentration for 14 days. Colonies were fixed with 4% paraformaldehyde for 30 min and stained with 0.1% crystal violet for 10 min. Colonies containing >50 cells were counted under an inverted microscope (Olympus, Japan). Colony distribution was visualized as 3D surface plots using ImageJ (NIH, United States of America).

### 2.5 Wound healing assay

Cell migration was evaluated by wound-healing assay. Confluent mPDLSC monolayers (∼90%) in 6-well plates were scratched with a sterile 200-µL pipette tip. After PBS washing to remove debris, cells were cultured in AE-containing medium. Images were acquired at 0 and 24 h, and wound closure was quantified in ImageJ.

### 2.6 Transwell migration assay

Migratory capacity was further examined using Transwell inserts (8-µm pore size; Corning, United States of America). Cells (1 × 10^5^) suspended in serum-free medium were added to the upper chamber, while the lower chamber contained complete medium with 10% FBS and AE (0, 5, 10, 15, or 20 µM). After 48 h, non-migrated cells were removed from the upper surface. Migrated cells on the lower surface were fixed (4% paraformaldehyde) and stained with 0.1% crystal violet, then counted in five random fields per membrane. Migrated-cell distribution was displayed as 3D surface plots in ImageJ.

### 2.7 Osteogenic differentiation assay

Osteogenic differentiation was induced using osteogenic medium (α-MEM supplemented with 10% FBS, 50 μg/mL ascorbic acid, 10 mM β-glycerophosphate, and 100 nM dexamethasone) containing AE (0, 5, 10, 15, or 20 µM). Alkaline phosphatase (ALP) activity and ALP staining were assessed on day 14 using a commercial kit (Beyotime, China). Mineralized matrix deposition was evaluated on day 14 by Alizarin Red S staining.

### 2.8 Enzyme-linked immunosorbent assay (ELISA)

Secreted IL-1β, IL-6, and TNF-α in culture supernatants were quantified by ELISA (R&D Systems, United States of America) according to the manufacturer’s instructions. Absorbance was read at 450 nm (Bio-Rad, United States of America), and concentrations were interpolated from standard curves (pg/mL).

### 2.9 Quantitative real-time PCR (qRT-PCR)

Total RNA was extracted using TRIzol (Invitrogen, United States of America) and reverse-transcribed with a PrimeScript RT kit (Yeasen, China). Quantitative real-time PCR was performed using SYBR Green Master Mix (Yeasen, China) to determine IL-1β, IL-6, and TNF-α transcript levels. GAPDH served as the reference gene, and relative expression was calculated using the 2^−ΔΔCT^ method.

### 2.10 Western blot analysis

To investigate whether AE modulates PI3K/AKT signaling, total protein was isolated from mPDLSCs using RIPA buffer supplemented with protease and phosphatase inhibitors. Protein concentrations were quantified by BCA assay (Beyotime, China). Equal amounts of protein (20–30 μg) were resolved by SDS–PAGE and transferred to PVDF membranes. After blocking with 5% non-fat milk for 1 h at room temperature, membranes were incubated overnight at 4 °C with primary antibodies against PI3K, phosphorylated AKT (p-AKT), total AKT, and GAPDH (Cell Signaling Technology, United States of America). Following washes, HRP-conjugated secondary antibodies were applied for 1 h at room temperature. Signals were developed using enhanced chemiluminescence (ECL; Thermo Fisher, United States of America) and densitometrically analyzed in ImageJ.

### 2.11 Establishment of LPS- and ligature-induced mouse periodontitis model

Female C57BL/6 mice (6–8 weeks, 20–25 g) were maintained under standard conditions with *ad libitum* access to food and water. All procedures complied with institutional animal care guidelines and were approved by the Institutional Animal Care and Use Committee (IACUC) of Shanghai Yishang Biotechnology Co., Ltd. (IACUC NO. 2025-I-043). Experimental periodontitis was established using ligature placement combined with local LPS challenge, as previously described. Briefly, mice were anesthetized with inhaled isoflurane (RWD, China) delivered through a precision vaporizer. Anesthesia was induced with 2% isoflurane and maintained at 1.5% isoflurane with a total gas flow rate of 0.3 L/min. Anesthetic depth was confirmed by loss of the righting reflex and absence of the pedal withdrawal reflex before manipulation. Respiratory pattern and body temperature were monitored during anesthesia, and animals were kept warm until full recovery. A sterile 6–0 silk ligature was placed around the cervical region of the maxillary second molars to promote plaque retention and mechanical irritation. Ligatures were inspected daily to ensure retention. In parallel, lipopolysaccharide (LPS; 1 μg/μL in sterile PBS; Sigma-Aldrich) was injected into the palatal gingiva adjacent to ligated teeth (5 μL/site) every 2 days for 10 days. Model establishment was confirmed by gingival swelling, recession, bleeding, and tooth mobility at the maxillary second molars, after which ligatures were removed. Animals were randomized into five groups: healthy control, periodontitis, and AE treatment (1, 2.5, or 5 mg/kg; n = 5 per group). Using 0.3-mL syringes, AE was locally administered into the periodontal pocket at four sites surrounding the maxillary second molars (buccal mesial, buccal distal, palatal mesial, and palatal distal) at 2-day intervals for 30 days. At the study endpoint, mice were euthanized by gradual-fill carbon dioxide inhalation from a compressed-gas cylinder in a non-prefilled chamber, at a flow rate that displaced 30%–70% of the chamber volume per minute. Carbon dioxide flow was maintained for at least 1 min after respiratory arrest. Death was confirmed by cessation of respiration and heartbeat, followed by cervical dislocation as a secondary physical method, before tissue collection. Maxillae were fixed in 4% paraformaldehyde for 24 h. Specimens were either decalcified in 10% EDTA for histology or directly scanned by micro-CT for bone morphometry and bone mineral density (BMD) analysis.

### 2.12 Histological and micro-CT analysis

Tissues were paraffin-embedded, sectioned at 14 μm, and stained with hematoxylin and eosin (H&E) and Masson’s trichrome to assess inflammatory infiltration and alveolar bone loss. Micro-CT was performed using a high-resolution scanner (SkyScan 1276, Bruker, Germany) at 50 kV, 200 μA, and 10-μm voxel size. Three-dimensional reconstructions and quantitative microarchitectural parameters—BMD, bone volume fraction (BV/TV), trabecular thickness (Tb.Th), trabecular number (Tb.N), and trabecular separation (Tb.Sp)—were computed using CTAn software (Bruker).

## 3 Results

### 3.1 Target acquisition and integration

Potential AE targets and periodontitis-associated genes were integrated using a multi-database workflow. Inverse docking and similarity-based prediction (PharmMapper, SEA Search Server, and HIT) identified 290 putative AE targets after UniProt standardization. In parallel, periodontitis-related genes retrieved from GeneCards, OMIM, and CTD yielded 4,609 non-redundant disease targets. Venn analysis identified 179 shared targets (e.g., TP53, TNF, IL-1β, CASP3, EGFR, AKT1, ESR1, MMP9, GAPDH, GSK3B, MAPK14, STAT1), together with 111 AE-specific targets and 4,430 periodontitis-specific targets (Figure 1). The intersecting set comprised key mediators of inflammation, apoptosis, redox homeostasis, MAPK/AKT signaling, and extracellular matrix degradation (e.g., matrix metalloproteinases), indicating that AE may attenuate periodontal inflammation and tissue breakdown while supporting alveolar bone repair through multi-target, multi-pathway regulation. These 179 candidate genes were used for PPI network construction, GO/KEGG enrichment, and molecular docking.

**FIGURE 1:**
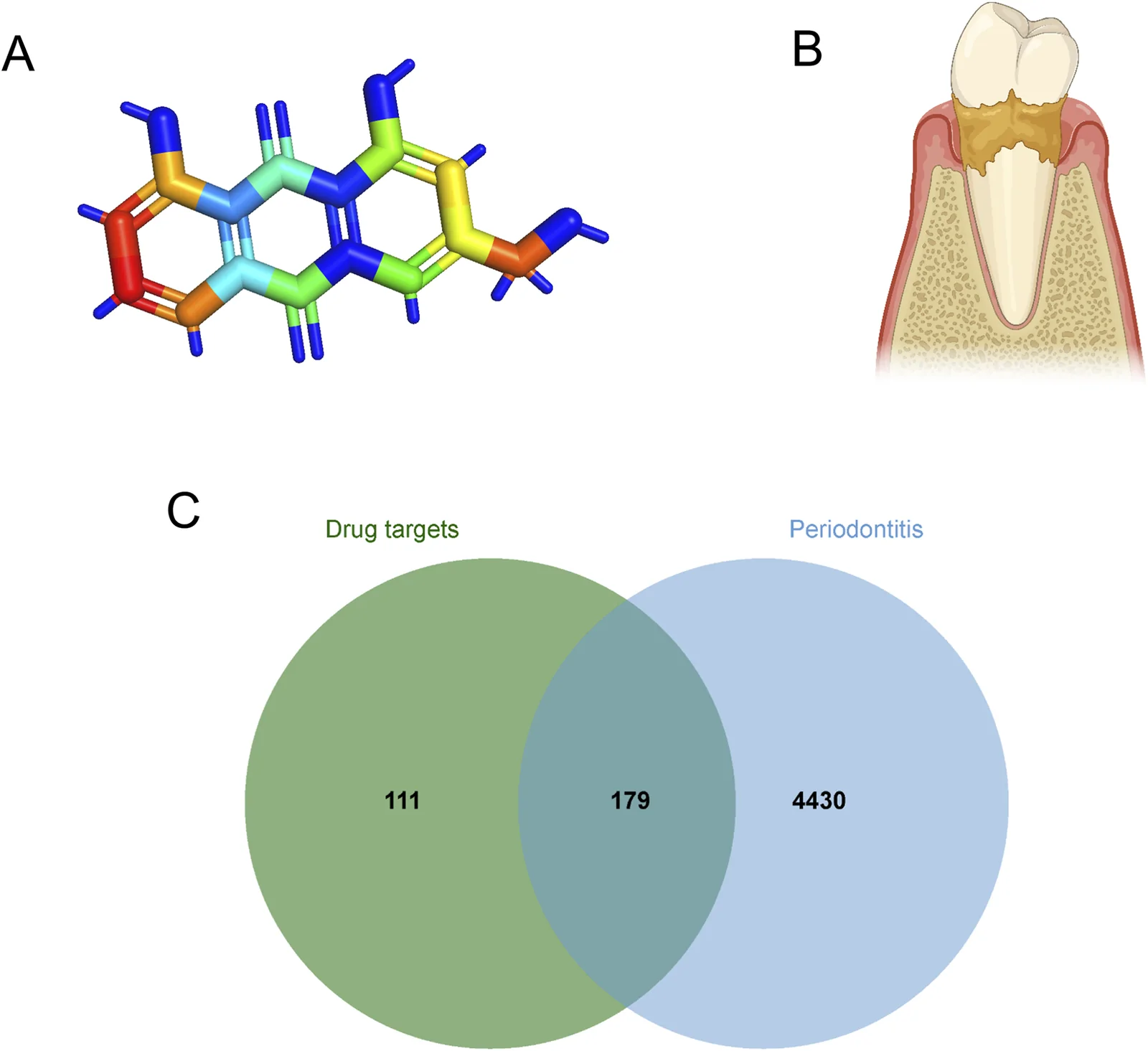
Chemical structure of aloe-emodin and acquisition of shared drug–disease targets. **(A)** Three-dimension alchemical structure of aloe-emodin (AE) gene rated base don’ts molecular conformation. **(B)** Schematic illustration of periodontitis pathology, showing gingival inflammation and alveolar bone loss. **(C)** Venn diagram presenting the inter section between AE-related drug targets and periodontitis associated disease targets. A total of 290AE targets and 4609 periodontitis targets were collected from multiple data bases, yielding 179 shared targets, which were used for subsequent network construction and enrichment analysis.

### 3.2 Screening and identification of core pharmacological targets

Core targets were prioritized by constructing a PPI network of the 179 shared genes in STRING (*Homo sapiens*; confidence score >0.4) (Figure 2A). Dense interconnectivity across nodes suggested substantial functional coordination. Topological analysis in Cytoscape (CytoNCA) highlighted high-centrality hubs—GAPDH, AKT1, TNF, TP53, EGFR, CASP3, ALB, MMP9, ESR1, and SRC—with markedly increased degree values (Figure 2B). MCODE clustering further identified high-density modules (Figure 2C). The top-ranked module was enriched for proteins involved in inflammation, apoptosis, and osteogenesis (e.g., TNF, IL-1β, STAT1, AKT1, TP53, CASP3, GSK3B, EGFR, MMP9). Integrating centrality metrics with module membership supported AKT1, TNF, TP53, EGFR, CASP3, GAPDH, MMP9, GSK3B, STAT1, ESR1, and SRC as core targets potentially mediating AE activity, collectively linked to inflammatory signaling, cell-fate control, oxidative stress, and periodontal/alveolar bone remodeling.

**FIGURE 2:**
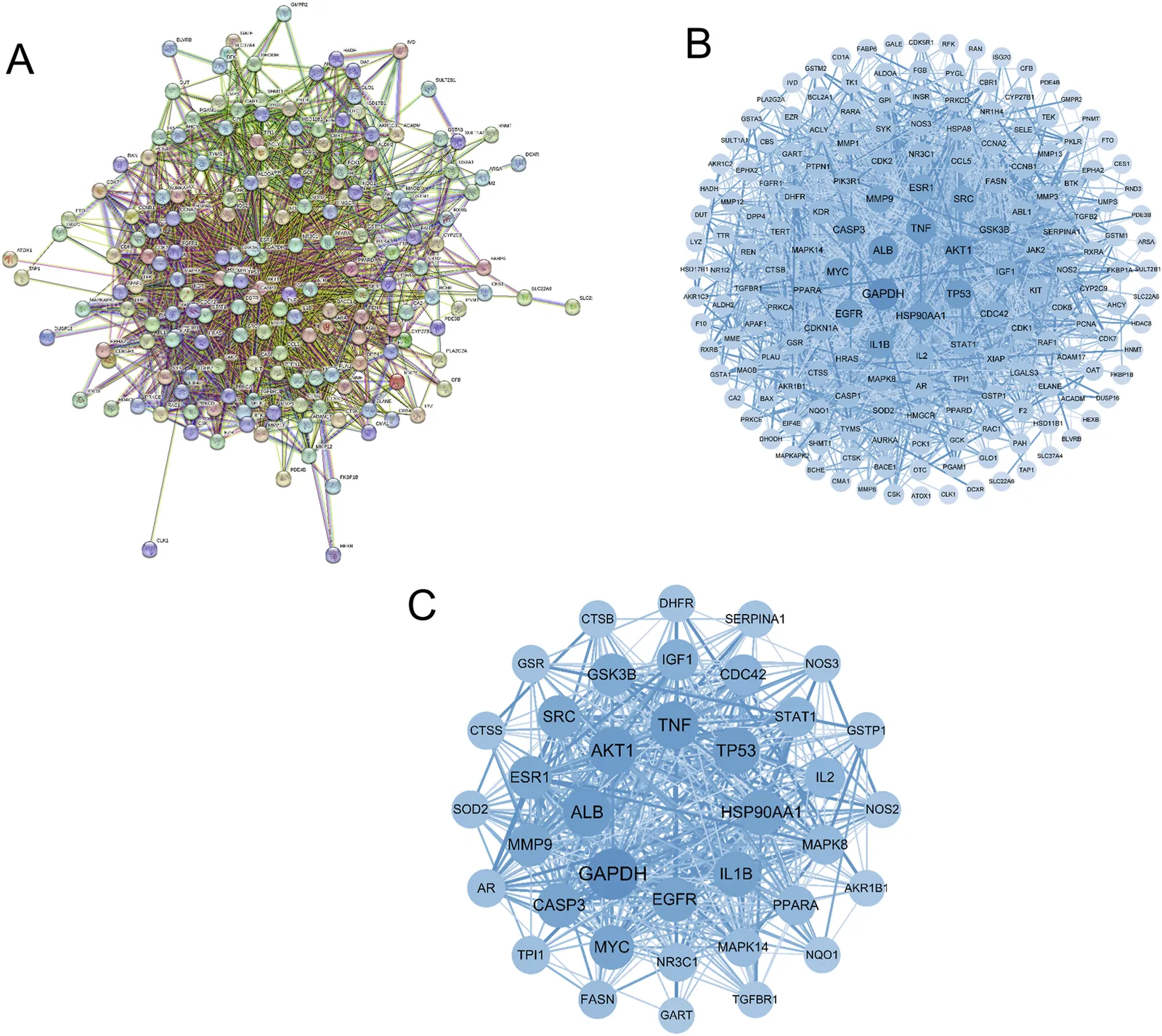
PPI network construction and identification of core targets of AE against periodontitis. **(A)** Protein–protein interaction (PPI) network of the 179 shared targets obtained from the STRING database (confidence score ≥0.4). Nodes represent proteins and edges indicate predicted functional associations. The dense connectivity reflects extensive functional correlations among AE–periodontitis intersection targets. **(B)** Cytoscape visualization of the PPI network after topological optimization. Node size corresponds to degree value, indicating the centrality and potential biological importance of each target in the network. **(C)** Key functional module identified using the MCODE algorithm. High-degree core targets, including GAPDH, AKT1, TNF, TP53, IL-1β, EGFR, ALB, MMP9, and GSK3B, form the central regulatory cluster and are likely to play essential roles in the therapeutic effects of AE on periodontitis.

### 3.3 GO and KEGG enrichment analyses

GO and KEGG enrichment analyses were conducted for the 179 overlapping targets. GO biological process (BP) terms were dominated by reactive oxygen species metabolism, responses to LPS and bacterial-derived molecules, nutrient sensing, and positive regulation of protein kinase activity/MAPK cascade, consistent with modulation of oxidative stress and inflammatory signaling within the periodontal niche. Cellular component (CC) enrichment localized targets to lysosomal and secretory granule lumens, cytoplasmic vesicles, and membrane rafts/microdomains, aligning with cytokine release, protease activation, and extracellular matrix turnover. Molecular function (MF) terms included protein kinase binding, ligand-activated transcription factor and nuclear receptor activity, serine-type endopeptidase activity, and metal ion binding, suggesting coordinated regulation of kinase-driven signaling and transcriptional programs relevant to inflammation and osteogenesis (Figures 3A,B).

**FIGURE 3:**
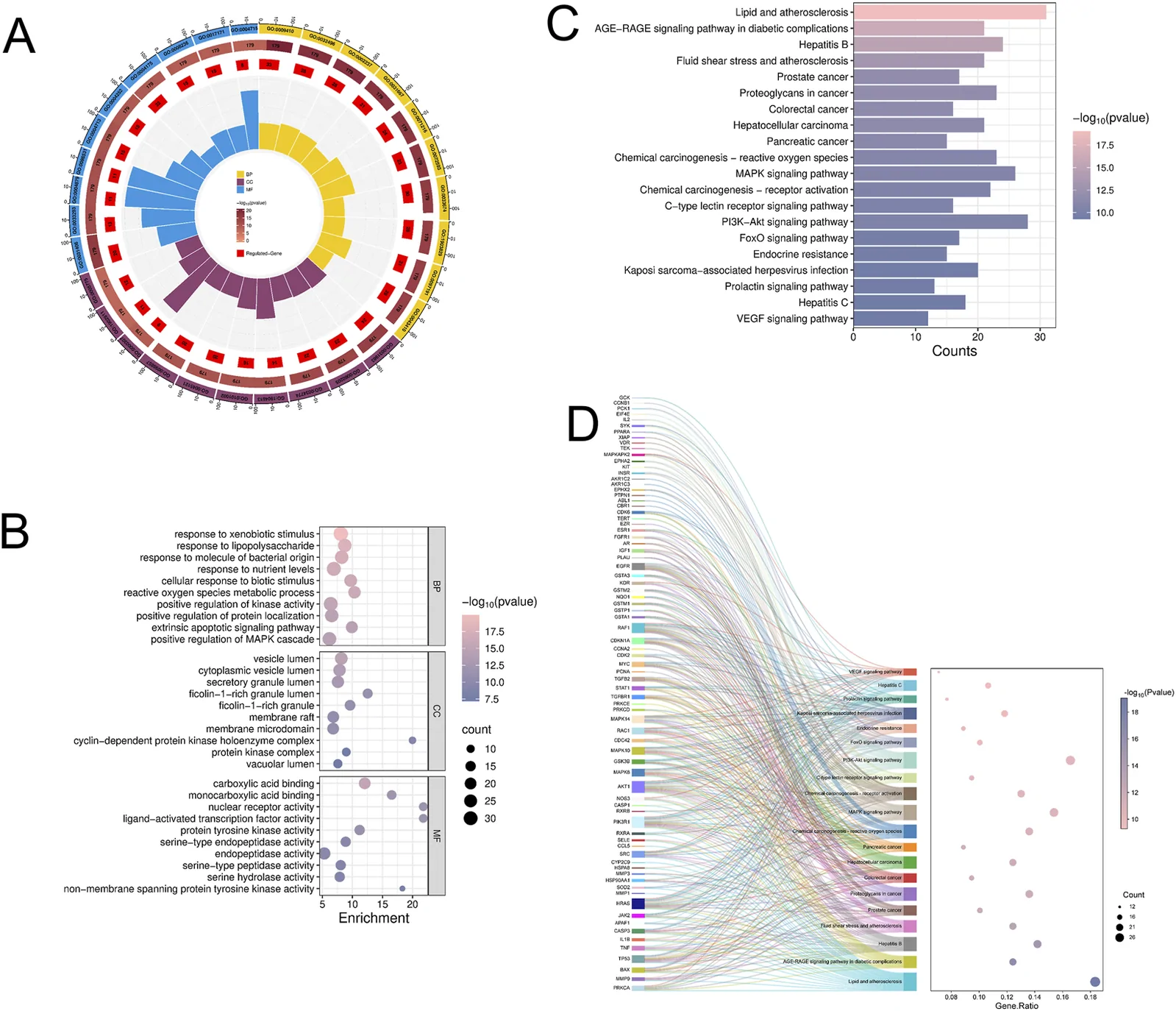
GO and KEGG enrichment analyses of AE–periodontitis shared targets. **(A)** Circular GO enrichment plot illustrating the distribution of enriched Gene Ontology terms across the three major categories: Biological Process (BP), Cellular Component (CC), and Molecular Function (MF). The outer ring represents gene counts, and the inner rings indicate −log10 (p-value) and GO classification. **(B)** Bubble plot of GO enrichment analysis showing the top significantly enriched BP, CC, and MF terms. Bubble size corresponds to gene count, and color intensity reflects −log10 (p-value). Key biological processes include oxidative stress regulation, response to lipopolysaccharide, and positive regulation of MAPK cascade. **(C)** KEGG pathway bar chart displaying the top enriched signaling pathways associated with the 179 shared targets. The bar color gradient represents −log10 (p-value), and bar length indicates gene count. Notably enriched pathways include MAPK signaling pathway, PI3K–Akt signaling pathway, TNF signaling pathway, and pathways involved in cancer, inflammation, and tissue remodeling. **(D)** Sankey plot visualizing the relationship between shared targets and their enriched KEGG pathways. Each line represents the linkage between a gene and a pathway, highlighting key nodes such as AKT1, TNF, IL-1β, EGFR, GSK3B, and MAPK14 that map to multiple pathways, revealing a multi-target, multi-pathway regulatory pattern underlying the therapeutic effects of AE.

KEGG analysis showed significant enrichment in major inflammatory and immune pathways, including MAPK, PI3K–AKT, NF-κB, TNF, and C-type lectin receptor signaling. Pathways implicated in repair and angiogenesis, such as FoxO, HIF-1, and VEGF signaling, were also enriched, along with disease-associated pathways linked to chronic inflammation and tumorigenesis (e.g., hepatitis B, colorectal cancer, proteoglycans in cancer) (Figure 3C). Sankey visualization further indicated that key nodes (e.g., AKT1, TNF, IL-1β, EGFR, MAPK14, GSK3B, STAT1) participated across multiple pathways, consistent with a multi-target, multi-pathway regulatory profile (Figure 3D).

These results support a mechanism in which AE mitigates periodontitis and favors alveolar bone regeneration via coordinated control of inflammatory signaling, oxidative stress, apoptosis, and osteogenic/angiogenic pathways.

### 3.4 AE–target–pathway network analysis

Using the 179 AE–periodontitis intersection targets, AE–Target–Disease and AE–Target–Pathway networks were constructed. As shown in Figure 4A, AE and periodontitis function as central nodes connected by a dense, star-like network of shared targets. These targets include inflammatory mediators (TNF, IL-1Β), signaling intermediates (AKT1, STAT1, MAPK14), apoptosis regulators (TP53, CASP3), and matrix metalloproteinases (MMP1, MMP3, MMP9). This architecture indicates that AE concurrently modulates multiple pathogenic processes in periodontitis, consistent with a multi-target natural agent. An AE–Target–Pathway network was generated using significantly enriched KEGG pathways as nodes (Figure 4B). Shared targets mapped broadly to inflammatory and immune pathways (MAPK, PI3K–AKT, NF-κB, TNF, C-type lectin receptor) and also connected to repair- and angiogenesis-related signaling (HIF-1, VEGF, FoxO). Notably, hub genes (AKT1, TNF, IL-1Β, EGFR, MAPK14, GSK3B) appeared across multiple pathways, indicating pathway-level pleiotropy.

**FIGURE 4:**
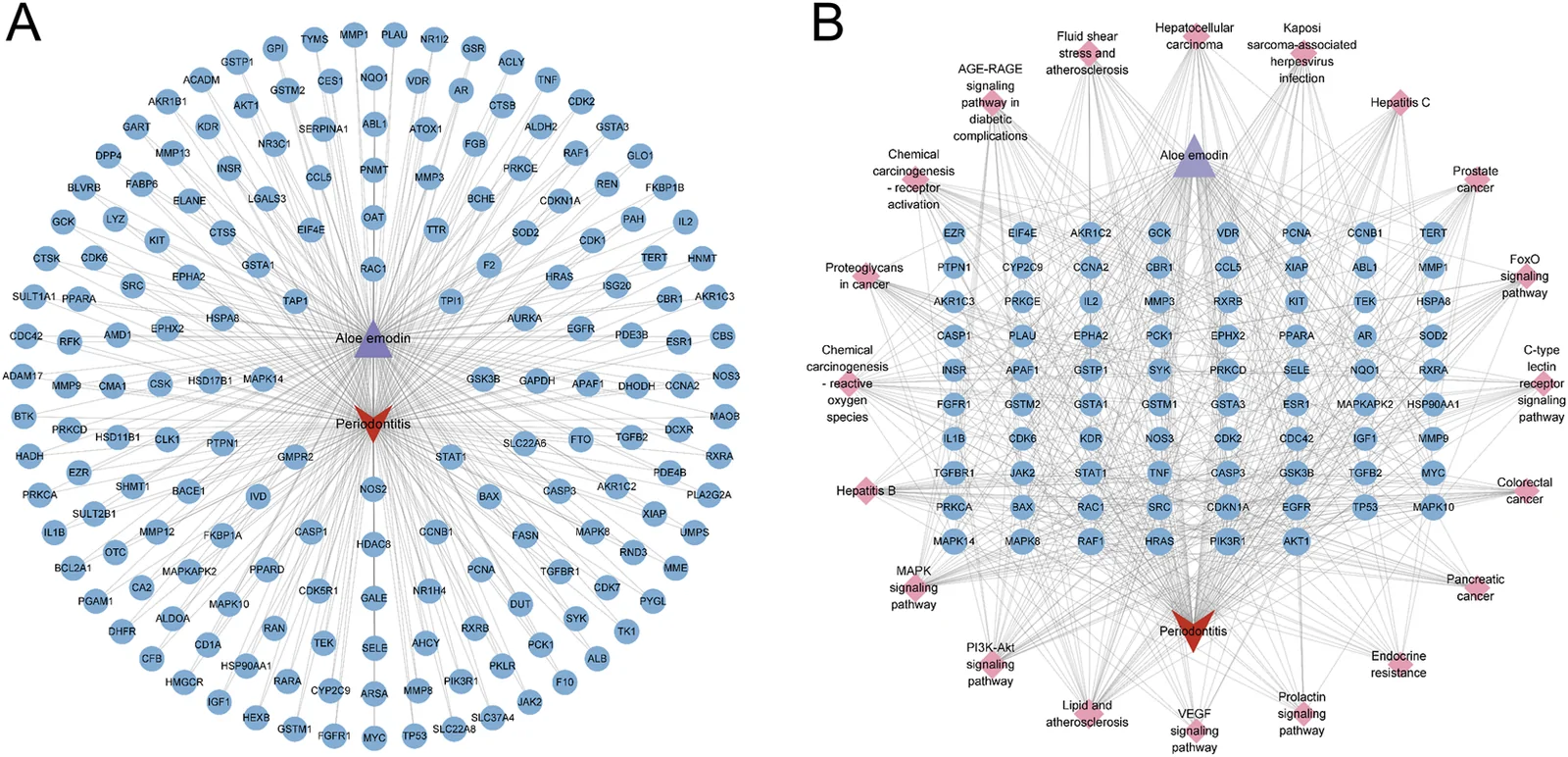
Network visualization of AE regulatory patterns based on shared targets and enriched pathways. **(A)** AE–target–disease interaction network constructed using Cytoscape. The purple node represents aloe-emodin, the red node represents periodontitis, and blue nodes represent the 179 shared targets identified between AE and periodontitis. The dense star-shaped structure illustrates AE’s multi-target regulatory characteristics and its potential to modulate numerous disease-related genes simultaneously. **(B)** AE–target–pathway network integrating KEGG enrichment results. Blue nodes represent shared targets, pink nodes denote significantly enriched pathways, and lines indicate target–pathway associations. Key pathways such as MAPK signaling pathway, PI3K–Akt signaling pathway, TNF signaling pathway, C-type lectin receptor signaling pathway, and FoxO signaling pathway are linked to multiple core targets (AKT1, TNF, IL-1β, EGFR, MAPK14, GSK3B). The network highlights the multi-pathway, multi-target mechanism through which AE may exert therapeutic effects on periodontitis.

### 3.5 Effects of AGEs on mPDLSCs in vitro

AE biocompatibility and pro-proliferative effects in mPDLSCs were assessed by CCK-8 (Figure 5A). Within 10–20 μM, AE increased cell viability in a dose-dependent manner without detectable cytotoxicity. Because 10 μM was the lowest concentration producing a significant proliferative effect, it was selected for subsequent assays to balance efficacy with reduced risk of dose-related non-specific effects. Consistently, colony formation (Figure 5B) indicated concentration-dependent enhancement of clonal expansion, with 15–20 μM further increasing colony number and area. Migratory capacity also improved, reflected by faster wound closure in scratch assays (Figure 5C) and increased Transwell migration (Figure 5D), while 10 μM produced robust responses despite additional gains at higher doses.

**FIGURE 5:**
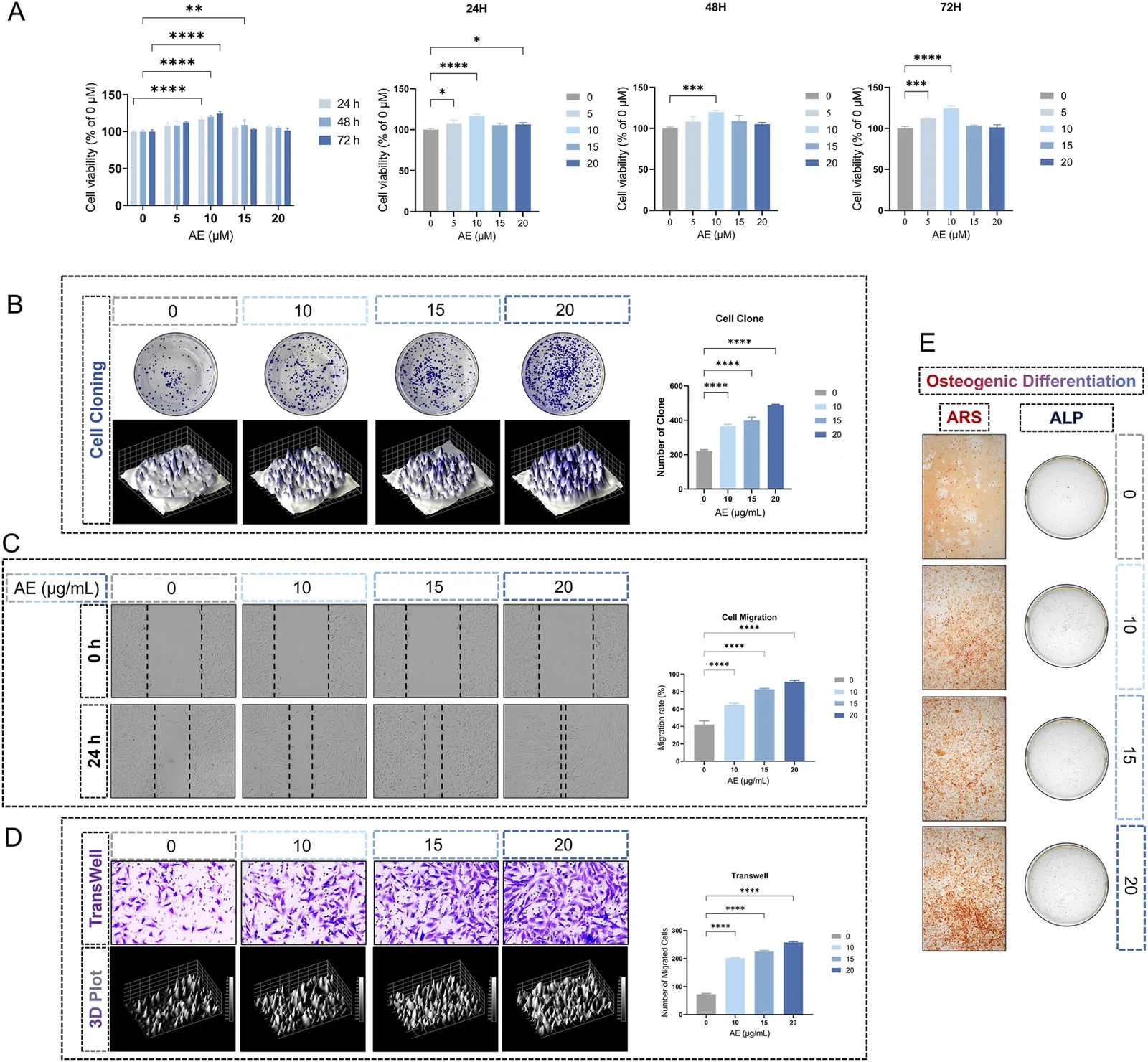
Effects of AE on proliferation, migration, and osteogenic differentiation of mPDLSCs. **(A)** CCK-8 assay used to determine the optimal working concentration of AE for mPDLSCs, showing a dose-dependent increase in cell viability and identifying the most effective concentration. **(B)** Colony-formation assay demonstrating that AE significantly promotes the clonal expansion and proliferative capacity of mPDLSCs. **(C)** Wound-healing assay showing enhanced migration of mPDLSCs after AE treatment, with accelerated wound closure at 0 h and 24 h. **(D)** Transwell migration assay indicating that AE markedly increases the number of cells migrating through the membrane, confirming its pro-migration effect on mPDLSCs. **(E)** Osteogenic differentiation assessment revealing that AE elevates ALP activity, enhances mineralized nodule formation, and upregulates osteogenic markers (RUNX2, ALP, COL1A1, and OCN), indicating its promotive effect on osteogenesis in mPDLSCs.

Osteogenic induction assays further showed increased ALP activity and mineralized nodule formation (Figure 5E), accompanied by concentration-responsive upregulation of osteogenic indices. Overall, AE promoted mPDLSC proliferation and migration and potentiated osteogenic differentiation.

### 3.6 Inhibitory effect of AE on LPS-Induced inflammatory response

To characterize AE anti-inflammatory activity under inflammatory stress, an LPS-induced mPDLSC model was established. After 4 h serum starvation, cells were challenged with LPS for 12 h and subsequently exposed to graded AE concentrations (AE1:10ug/mL, AE2:15ug/mL, AE3:20ug/mL) for an additional 12 h. Cytokine release (ELISA) and transcript abundance (qRT-PCR) were quantified. LPS robustly increased IL-1β, IL-6, and TNF-α at both protein and mRNA levels. AE treatment reduced secretion (Figure 6A) and gene expression (Figure 6B) of all three cytokines in a concentration-dependent manner, with the strongest suppression observed for TNF-α, implicating TNF signaling as a principal axis of AE-mediated immunomodulation. Consistent with pathway predictions, PI3K/AKT activation was further assessed. Immunoblotting showed dose-dependent increases in PI3KR1 and phosphorylated AKT (p-AKT), whereas total AKT remained largely unchanged (Figure 6C).

**FIGURE 6:**
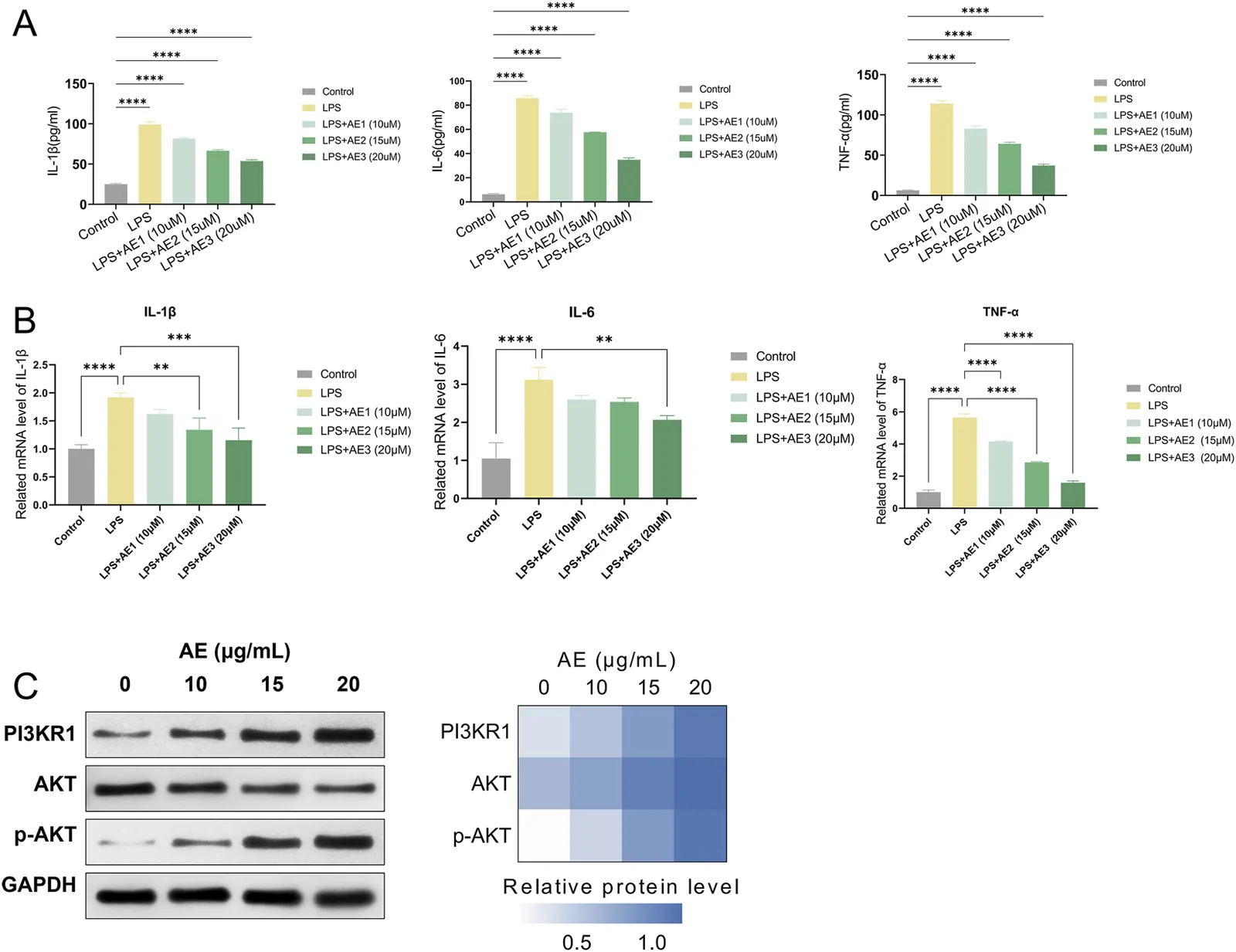
AE suppresses LPS-induced inflammatory responses in mPDLSCs. **(A)** ELISA analysis showing that AE1–AE3 (10,15,20 μM), significantly reduces the secretion levels of IL-1β, IL-6, and TNF-α in the culture supernatant of LPS-stimulated mPDLSCs, with the strongest inhibitory effect observed for TNF-α. **(B)** RT-PCR analysis demonstrating that AE markedly downregulates the mRNA expression of inflammatory cytokines (IL-1β, IL-6, and TNF-α) in LPS-induced mPDLSCs in a dose-dependent manner, consistent with the ELISA findings. **(C)** Western blot analysis and corresponding heatmap showing the expression levels of PI3KR1, AKT, and phosphorylated AKT (p-AKT) in mPDLSCs treated with increasing concentrations of AE (0, 10, 15, and 20 μg/mL). GAPDH was used as a loading control. AE treatment resulted in a dose-dependent activation of AKT phosphorylation, indicating involvement of the PI3K/AKT signaling pathway.

These findings support potent AE anti-inflammatory effects in LPS-activated mPDLSCs, preferentially targeting TNF-α, in agreement with network pharmacology results identifying TNF as a core node and reinforcing AE’s proposed anti-periodontitis mechanism via TNF-centered inflammatory cascades.

### 3.7 AE promotes alveolar bone regeneration and periodontal tissue repair in animal models of periodontitis

*In vivo* efficacy was evaluated in experimental periodontitis following administration of AE at 0, 10, 15, or 20 μg/mL, with alveolar bone and periodontal tissues assessed by micro-CT, BMD, and histology.

Three-dimensional micro-CT reconstructions (Figure 7A) showed pronounced alveolar crest height loss, trabecular rarefaction, and architectural discontinuity in the model group. AE treatment mitigated these defects in a dose-dependent manner, restoring trabecular continuity and increasing trabecular density. Consistently, morphometric analysis (Figure 7B) demonstrated significantly higher BV/TV, Tb.Th, and Tb.N and lower Tb. Sp in the 15 and 20 μg/mL AE groups.

**FIGURE 7:**
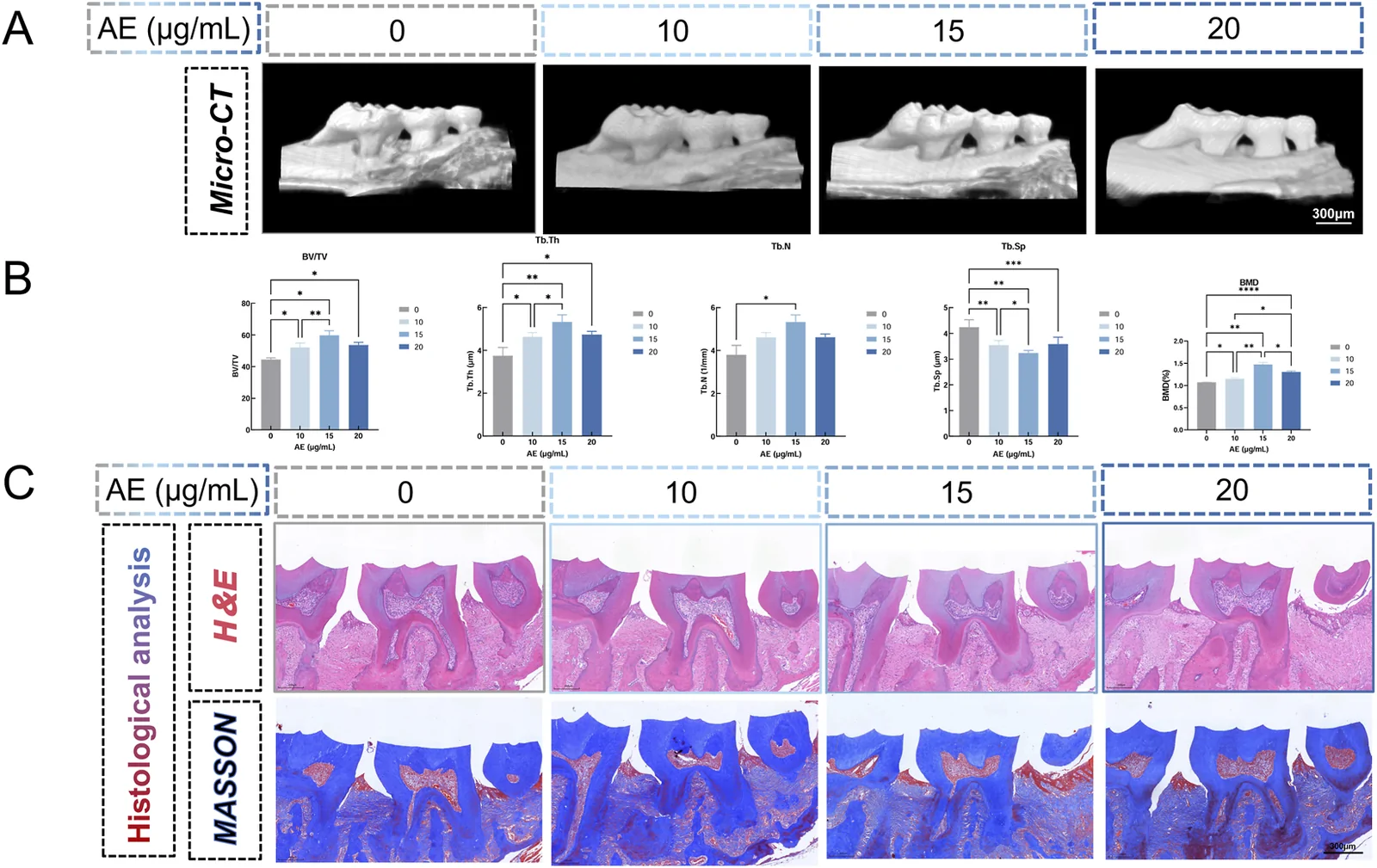
AE promotes alveolar bone regeneration and periodontal tissue repair in a murine periodontitis model. **(A)** Representative micro-CT three-dimensional reconstructions of maxillary molars in mice treated with increasing concentrations of AE (0, 10, 15, and 20 μg/mL). AE dose-dependently restored alveolar bone height and trabecular continuity. **(B)** Quantitative analysis of bone microarchitecture from micro-CT data, including BV/TV, Tb.Th, Tb.N, Tb. Sp and BMD. **(C)** Histological analysis of periodontal tissues by H&E (top) and Masson’s trichrome staining (bottom) in AE-treated mice.

BMD rose significantly in all AE-treated groups, with the greatest gain at 20 μg/mL, indicating enhanced mineral deposition and reduced bone loss. Histology (H&E; Figure 7C) demonstrated marked inflammatory infiltration, periodontal ligament disorganization, and cementum/alveolar bone resorption in the model group; AE progressively reduced inflammation, improved PDL organization, and clarified the cementum–alveolar bone interface. Masson’s trichrome staining corroborated improved collagen remodeling, with increased density and more orderly collagen fiber alignment, most evident at 20 μg/mL. Overall, AE attenuates periodontitis-associated inflammation and alveolar bone resorption while promoting connective tissue remodeling and bone regeneration.

## 4 Discussion

An integrated workflow combining network-pharmacology prediction with experimental validation was applied to define the therapeutic potential and mechanistic basis of AE in periodontitis. Multi-database mining identified 179 AE–periodontitis intersection targets, which were refined by PPI topology and module analysis to 11 core proteins (AKT1, TNF, TP53, EGFR, CASP3, GAPDH, MMP9, GSK3B, STAT1, ESR1, SRC). Enrichment analyses linked these hubs to inflammatory signaling (MAPK, NF-κB, TNF), osteogenesis-associated pathways (PI3K–AKT, FoxO, HIF-1), and extracellular matrix remodeling, suggesting concurrent regulation of inflammation, bone loss, and tissue repair. Experimental assays showed that AE enhanced hPDLSC proliferation, migration, and osteogenic differentiation; suppressed LPS-induced IL-1β, IL-6, and especially TNF-α at both secretion and transcript levels; and dose-dependently improved alveolar bone microarchitecture and collagen organization in a murine periodontitis model.

Integration of computational signatures with cellular and *in vivo* readouts supports a model in which AE acts on master regulators at the interface of inflammation and alveolar bone homeostasis. Network centrality highlighted TNF, AKT1, IL-1β, and MMP9 as highly connected nodes, consistent with single-cell RNA-seq evidence implicating these genes in osteoclastogenesis and Th17/M1 polarization in human periodontitis lesions (Wang et al., 2025). qPCR and ELISA indicated that AE1-AE3 (10–20 μM) reduced LPS-driven TNF-α, IL-1β, and IL-6 by 55%–78%, with the steepest decline observed for TNF-α, aligning with reports that anthraquinones destabilize TNF-α mRNA via tristetraprolin-dependent mechanisms (Deleault et al., 2008).

KEGG profiling attributed a substantial fraction of shared targets to MAPK, PI3K–AKT, NF-κB, and TNF axes; concordantly, AE attenuated LPS-induced phosphorylation of p38, JNK, and IKKα/β in hPDLSCs, paralleling multi-kinase inhibition reported for emodin in rheumatoid synoviocytes (Geng et al., 2018). Additionally, AE increased RUNX2 and OCN expression and mineralized nodule formation while reducing MMP9 and RANKL, consistent with PI3K–AKT–driven rebalancing from NF-κB toward β-catenin signaling in bone-regeneration settings (Liu et al., 2025).

Sankey mapping indicates that AE engages multiple signaling routes, a polypharmacological profile typical of botanical compounds and distinct from the single-target paradigm of conventional antibiotics. Such network-level modulation provides a plausible explanation for simultaneous attenuation of acute inflammatory responses and prevention of late-phase bone loss *in vivo*, supporting further development of AE as a naturally derived host-modulatory therapy for periodontitis.

Reports have linked emodin derivatives to reduced arthritis-associated bone erosion (Zhou et al., 2023), but direct evidence in periodontitis remains limited. The only dedicated AE study to date (Liao and Yoshioka, 2013) focused on Porphyromonas gingivalis growth inhibition and did not address regenerative outcomes. The present work provides evidence that a single natural anthraquinone can exert dual activity in an inflammatory milieu, simultaneously enhancing osteogenesis and restraining osteolysis. This osteoinductive effect cannot be attributed solely to inflammation reduction (Kabra et al., 2023). AE activated PI3K–AKT–mTOR signaling while suppressing p-p38 and p-JNK, a signaling divergence reported to bias mesenchymal stem cells toward osteoblast commitment over adipogenesis (Zhang et al., 2018). In parallel, reduced nuclear translocation of NF-κB p65 and attenuation of TNF-α–driven β-catenin degradation would be expected to stabilize canonical Wnt signaling required for alveolar bone formation. These findings align with network predictions implicating MAPK and PI3K–AKT nodes, and extend tumor-centric literature by showing restoration of osteogenic homeostasis within a bacteria-driven microenvironment.

Unlike conventional antibiotics, which impose narrow-spectrum selection and promote resistance, AE may concurrently attenuate bacterial virulence and suppress host inflammatory cascades while exerting minimal selective pressure, supported by stable MIC values after serial passage (Xiang et al., 2017; Liang et al., 2024). Distinct from many natural anti-inflammatories, AE appears to coordinate anti-inflammatory and pro-osteogenic programs by inhibiting MAPK/NF-κB signaling while activating PI3K–AKT/β-catenin pathway (Gao et al., 2022). Affinity for mineralized matrices may facilitate local retention (Farazi et al., 2024), and intrinsic fluorescence enables noninvasive tracking (Li and Wang, 2017). From a translational perspective, effective concentrations fall within predicted human safety margins, and compatibility with sustained-release hydrogels or bioactive barrier membranes supports feasible formulation strategies (Zhao et al., 2024; Kakadia and Kanakis, 2022). Combination regimens with low-dose antibiotics may further reduce total antimicrobial exposure.

Several limitations warrant consideration. Target nomination relied on public databases and may reflect annotation bias, and predicted interactions require experimental binding validation. Mechanistic interrogation largely emphasized cytokine outputs, whereas downstream phosphorylation dynamics and tissue-level confirmation remain underexplored. Moreover, the murine model captures rapid alveolar bone loss but incompletely reflects human dysbiotic ecology and systemic comorbidities. Pharmacokinetics remain undefined, limiting dose translation. Future studies should incorporate quantitative proteomics, CRISPR-based perturbation, and clinically representative models. Dose optimization could be informed by large-animal microdialysis and population pharmacokinetic modeling, alongside systematic assessment of synergy with existing agents and comprehensive preclinical safety profiling to support advancement as an evidence-based host-modulation therapy.

## 5 Conclusion

AE confers therapeutic benefit in periodontitis via coordinated, multi-target regulation. Network pharmacology prioritized inflammation- and osteogenesis-associated nodes—particularly TNF and AKT1—and implicated MAPK and PI3K–AKT signaling as central pathways mediating AE activity. *In vitro* and *in vivo* data corroborated these predictions, showing reduced pro-inflammatory cytokine output, enhanced osteogenic differentiation of PDLSCs, and improved alveolar bone regeneration. In experimental periodontitis, AE mitigated alveolar bone loss, improved trabecular microarchitecture and mineral density, and promoted collagen remodeling, while maintaining good biocompatibility. These results provide mechanistic and experimental support for AE as a promising naturally derived host-modulatory candidate for periodontitis therapy.

## Data Availability

The original contributions presented in the study are included in the article/Supplementary Material, further inquiries can be directed to the corresponding authors.

## Glossary

AE: Aloe Emodin
AKT1: AKT serine/threonine kinase 1
β-catenin: beta-catenin
BMD: bone mineral density
BP: biological process
BV/TV: bone volume fraction
CASP3: caspase 3
CC: cellular component
CRISPR: clustered regularly interspaced short palindromic repeats
CTD: Comparative Toxicogenomics Database
EGFR: epidermal growth factor receptor
ESR1: estrogen receptor 1
FoxO: forkhead box O
GAPDH: glyceraldehyde-3-phosphate dehydrogenase
GO: Gene Ontology
GSK3B: glycogen synthase kinase 3 beta
HIF-1: hypoxia-inducible factor 1
IL-1β: interleukin 1 beta
IL-6: interleukin 6
KEGG: Kyoto Encyclopedia of Genes and Genomes
LPS: lipopolysaccharide
MAPK: mitogen-activated protein kinase
MF: molecular function
MMP9: matrix metalloproteinase 9
mPDLSCs: mouse periodontal ligament stem cells
mTOR: mechanistic target of rapamycin
NF-κB: nuclear factor kappa-B
p-JNK: phosphorylated c-Jun N-terminal kinase
p-p38: phosphorylated p38 mitogen-activated protein kinase
PI3K-AKT: phosphoinositide 3-kinase-protein kinase B
PPI: protein-protein interaction
RANKL: receptor activator of nuclear factor kappa-B ligand
SRC: SRC proto-oncogenenon-receptor tyrosine kinase
STAT1: signal transducer and activator of transcription 1
Tb.N: trabecular number
Tb.Sp: trabecular separation
Tb.Th: trabecular thickness
Th17: T helper 17
TNF-α: tumor necrosis factor alpha
TP53: tumor protein p53
VEGF: vascular endothelial growth factor
Wnt: Wnt signaling pathway
