# Supplementary material for: Unraveling the multitarget mechanisms of aloe emodin against periodontitis: a synergy of network pharmacology and experimental validation
Source: Front Pharmacol. 2026 Oct 20;17:1944429. doi: 10.3389/fphar.2026.1944429 (PMC13727639; doi:10.3389/fphar.2026.1944429)
Supplement: Supplementary file 1 [file Image1.pdf]

The caption of supplementary figure：

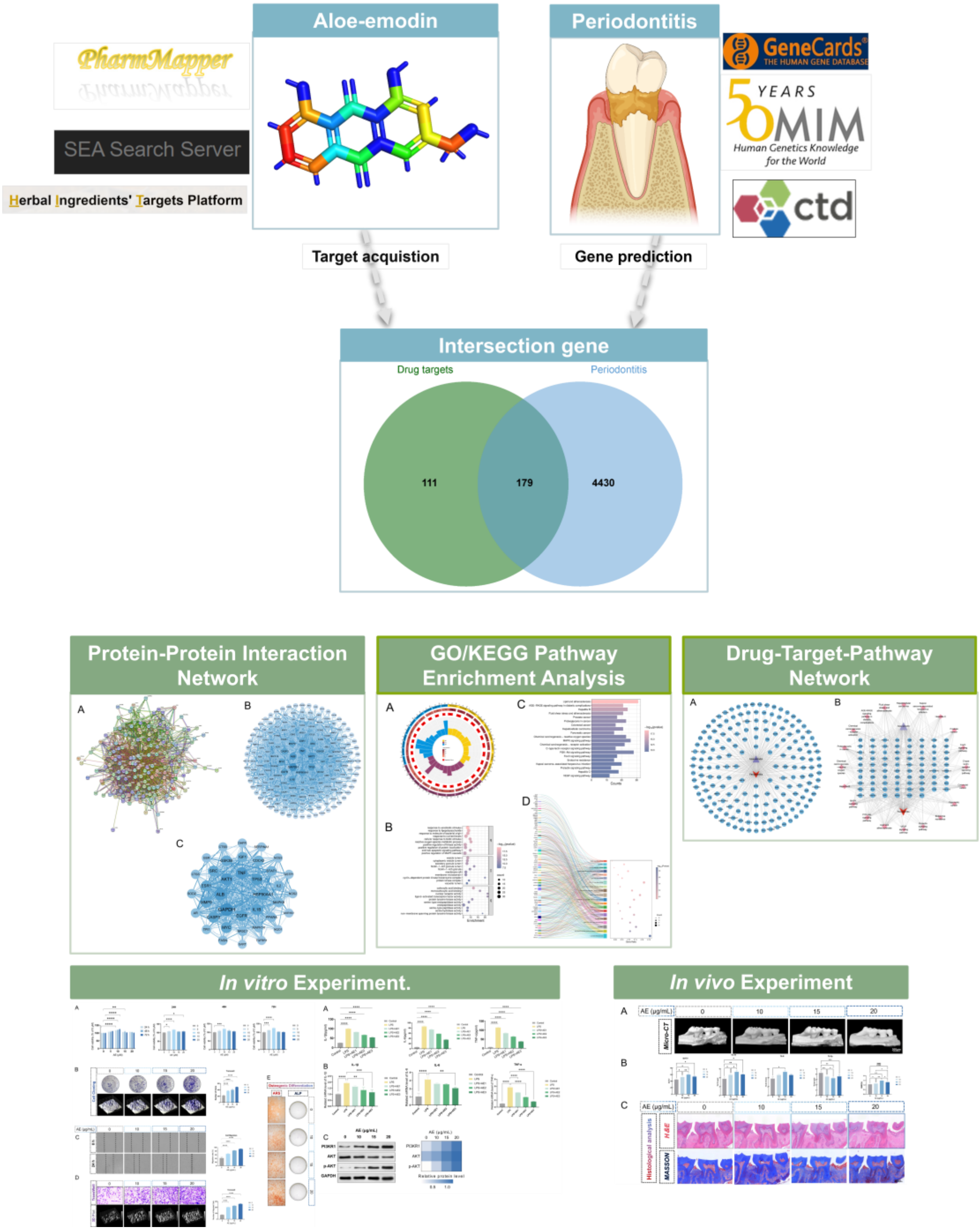

S1 graphical abstract
